# Disease of the Vermiform Appendix

**Published:** 1894-01-27

**Authors:** 


### DISEASES OF THE VERMIFORM APPENDIX.

A knowledge of the anatomy of the vermiform appendix is of interest to the physician and surgeon in its relations to the pathology, clinical history, and treatment of the morbid conditions of this appendage. Dr. J. D. Bryant has recorded[1] the results of an examination of the appendix in 150 autopsies conducted at Bellevue Hospital. His investigations may be summarised as follows:—

1. *Length of Appendix.*—The average length was, in the male $3\frac{1}{2}$ inches, in the female 3 1-10 inches.

2. *Origin of the Appendix.*—In one-half of the cases of both sexes the established point of origin of the vermiform appendix would appear to be one inch below the ileo-cæcal valve, and on the posterior surface of the cæcum. Hence finger point pressure, when directed from without, will invade the origin of the vermiform appendix from the cæcum in not less than 96 per cent. of all male cases.

3. *Diameter of Appendix.*—From an examination of twenty cases of each sex selected from the entire list indiscriminately, it appears that the average diameter in the male is about one-tenth of an inch greater than that of the female. The average diameter, based on the examination of these forty cases, irrespective of the presence in them or not of foreign matters, was found to be four-sixteenths of an inch.

4. *Contents of the Appendix.*—The greater the diameter of the appendix, within apparently normal limits, the greater the probability of the presence within it of fæcal or other foreign matter. From the examination of one hundred and twenty-four cases it appears (*a*) that 67 per cent. of the entire series contained abnormal material; (*b*) that abnormal material is met with more frequently in the appendix of the male (70 per cent.) than in that of the female (56 per cent.); (*c*) that fæcal matter, either soft or hard, is present more frequently in the appendix in both sexes than any other material; (*d*) that in no instance were there other than fæcal substances or the products dependent on inflammation present in these cases—grape seeds and bodies foreign to the intestine were not found at all; (*e*) that the presence of abnormal material in these cases (124) happened less frequently (67 per cent.) than is commonly stated; (*f*) that abnormal material is found chiefly between the ages of thirty and fifty years.

5. *Location and Direction of the Appendix.*—It is described as "directed onwards" in 24 per cent. of the male and 27 per cent. of the female cases. It was located behind the cæcum in the peritoneal cavity in about 20 per cent. of the male and 25 per cent. of the female cases. It extended into the true pelvis in about 14 per cent. of the male and in 7½ per cent. of the female cases.

6. *Mesenteric Attachments of the Appendix.*—In 40 per cent. of cases noted the appendix was "free," in that one-half or more of its length was surrounded entirely by peritoneum. The remaining cases had mesenterices of various lengths.

The subject of when and by what mode to operate in cases of appendicitis is one that has received a great deal of attention at the hands of surgeons, but cannot be said to be definitely settled. On this point Dr. Duncan[2] considers there are three varieties of the disease, which we may call the diffuse, the localised, and the relapsing. In the first, with or after more or less indication of origin in the region of the cæcum, there is speedily developed a general peritonitis which may remain adhesive and subsequently subside, or may pass quickly into the purulent condition, and then, if left to itself, it inevitably ends in death. The general principles by which one should be guided are sufficiently plain. The moment it is ascertained that the peritonitis is purulent, laparotomy must be performed; and again a patient ought not to be allowed to succumb to the disease without an operative attempt to relieve him of the origin of his danger. Dr. Duncan admits that the mode of arriving at a knowledge of these points involves the most anxious consideration of the most minute details in each individual case. In the second, the localised variety, the guide to operation is the presence of pus; but here again the principle is easy, but the practice by no means so simple, although it is better to err on the side of operating too soon than to run the risk of the abscess opening into a viscus or the general cavity of the peritoneum. In the third, or relapsing variety, no hard and fast rule can be laid down, but one must be guided largely by the circumstances of the patient as well as the severity of the case. Mr. Treves considers[3] that in relapsing appendicitis the more important circumstances which would justify an operation are as follow: (1) The attacks have been very numerous; (2) the attacks are increasing in frequency and severity; (3) the last attack has been so severe as to place the patient's life in considerable danger; (4) the constant relapses have reduced the patient to the condition of a chronic invalid, and have rendered him unfit to follow any occupation; (5) owing to the persistence of certain local symptoms during the quiescent period there is a probability that a collection of pus exists in or about the appendix.

It may be safe to argue that the pain and distress involved by the operation will be less than that attending any but a slight attack, and that the risk of the procedure is less than that associated with an outbreak of typhlitis considered generally. Treves' method of dealing with the appendix is, whenever possible, to make a circular cut through the peritoneum just on the distal side of the spot at which it is intended to sever the process. The peritoneum thus freed is turned back, as is the skin in a circular amputation. The appendix is cut across at the line of the reflected peritoneum. The mucous membrane which is present is scraped away with a sharp spoon. The muscular wall of the appendix is then brought together by means of a continuous No. 1 silk braid. Over the stump thus formed the reflected peritoneum is drawn, and secured in place by means of a few points of Lembert's suture. This is not always possible. The tube may have to be occluded by means of a single ligature, but an attempt should always be made to give to the stump a covering of peritoneum. The patient should remain in bed for twenty-one days. Dr. Ashton thinks it desirable to operate in almost any form of appendicitis, and formulates[4] the following rules:—

(1) That we must consider all cases of appendicitis as being imminently dangerous to life from the beginning of the attack, as there are no means of determining the exact pathological conditions present at the seat of disease; (2) that operative interference is indicated in mild cases, if medicinal treatment fails, within twenty-four hours, to produce a decided improvement in the symptoms; (3) that all cases of recurrent appendicitis should be operated upon as soon as the diagnosis is clear; (4) that increasing pain in the right iliac fossa, rapid pulse, continued elevation of temperature, tumour, and tympanites are conditions indicating immediate operation; (5) that it is unsafe to wait before operating for the development of symptoms indicating gangrene or perforation of the appendix, pus, bowel obstruction, or peritonitis.

Dr. McBurney has recorded[5] a case in which recovery from a general septic peritonitis had been secured by *abdominal incision, copious irrigation of the peritoneal cavity, and subsequent drainage.* On the subject of drainage, Dr. McBurney states that he seldom uses tubes where he can wipe out or wash out, cleaning out the cavity, which can afterwards be packed with gauze, and where the presumption is that the fluid effused will not be greater than the gauze can absorb in its meshes and hold until it is changed. Where, however, a large quantity of fluid has been thrown into the peritoneal cavity, and a very considerable quantity of it does not come out, it will find its way into the pelvis in such quantities that gauze will not be sufficient to absorb all of it. In such cases he would introduce a double drainage tube, and remove it as soon as that extra quantity of fluid has been got rid of.

[1] Annals of Surgery, Feb. 1893. [2] Edin Med. Journ., Aug., 1893. [3] Brit. Med. Journ., Ap. 22, 1893. [4] Therapeutic Gazette, Dec. 15, 1892. [5] Annals of Surgery, July, 1893.

